# Reductions in the United Kingdom's Government Housing Benefit and Symptoms of Depression in Low-Income Households

**DOI:** 10.1093/aje/kww055

**Published:** 2016-09-08

**Authors:** Aaron Reeves, Amy Clair, Martin McKee, David Stuckler

**Keywords:** depression, housing, mental health, natural experiment

## Abstract

Housing security is an important determinant of mental ill health. We used a quasinatural experiment to evaluate this association, comparing the prevalence of mental ill health in the United Kingdom before and after the government's April 2011 reduction in financial support for low-income persons who rent private-sector housing (mean reduction of approximately £1,220 ($2,315) per year). Data came from the United Kingdom's Annual Population Survey, a repeated quarterly cross-sectional survey. We focused our analysis on renters in the private sector, disaggregating data between an intervention group receiving the government's Housing Benefit (*n* = 36,859) and a control group not receiving the Housing Benefit (*n* = 142,205). The main outcome was a binary measure of self-reported mental health problems. After controlling for preexisting time trends, we observed that between April 2011 and March 2013, the prevalence of depressive symptoms among private renters receiving the Housing Benefit increased by 1.8 percentage points (95% confidence interval: 1.0, 2.7) compared with those not receiving the Housing Benefit. Our models estimated that approximately 26,000 (95% confidence interval: 14,000, 38,000) people newly experienced depressive symptoms in association with the cuts to the Housing Benefit. We conclude that reducing housing support to low-income persons in the private rental sector increased the prevalence of depressive symptoms in the United Kingdom.

***Editor's note:****An invited commentary on this article appears on page 430**, and the authors’ response appears on page 434*.

Housing provides shelter and security, protecting health and well-being; but when that security becomes uncertain, health (mental health in particular) is undermined. There is a large body of evidence documenting how difficulties in obtaining affording housing are associated with greater risks of depression (1–3), anxiety (4, 5), weight gain (6), visits to general practitioners (7), and suicide (8). However, whether the association between unaffordable housing and mental ill health is causal remains unclear (1, 9). Previous studies examining this relationship have been criticized for failing to address potential confounding (such as preexisting mental health or substance use problems) and have not identified why some households suddenly begin struggling to keep up with existing housing commitments. Housing may become unaffordable because of rising costs, such as increases in mortgage interest rates or rent, or because of a reduction in income, potentially through job loss, divorce, or welfare reductions (10). Earlier work has been unable to disentangle these drivers of unaffordable housing, which may, in turn, affect health to differing degrees.

To examine this question, we took advantage of a “natural policy experiment” that occurred when the United Kingdom's 2010–2015 Coalition government reduced incomes among low-wage renters by implementing large cuts to the Local Housing Allowance (LHA), a program which provides funds for tenants who rent housing in the private sector. Starting in 2010, the Coalition government embarked on a major austerity program, with the stated goal of reducing the nation's budget deficit. While much of the discussion of this policy has been dominated by macroeconomic considerations, public health professionals have argued that these policies will damage health (11–13).

Reductions in the Housing Benefit (HB) made one of the largest single contributions to the overall cut in spending (14). After pensions, the HB is the single largest welfare expenditure in the United Kingdom, costing the government approximately £24 billion ($31 billion) every year (14). Starting in April 2011, the Coalition government reduced funding for the LHA by £1.6 billion ($2.1 billion) (15–17). This did 2 things. First, it reduced the payment from the median value of local market rent to the 30th percentile. Second, it capped the amount of money households could receive at £250 ($326) per week for 1 bedroom, £290 ($378) per week for 2 bedrooms, £340 ($443) per week for 3 bedrooms, and £400 ($522) per week for 4 or more bedrooms. These policies were applied to both new and existing LHA claimants, from the anniversary date of their claim. The Institute for Fiscal Studies estimated that the average loss of income for recipients was £1,220 ($2,315) per year, affecting about 1.35 million individuals and potentially tipping 27,000–54,000 children into severe poverty (18).

This policy intervention, which was outside the control of academic researchers, created a “natural experiment.” Natural experiments are exogenous changes occurring in everyday life that assign people to either an intervention group or a control group through a process which is random or approximately random, such as winning the lottery or the timing of a policy intervention (19–21). The changes in the LHA therefore created a natural policy experiment among private renters, reducing incomes among those receiving the HB after April 2011 but not among renters in the private sector who were not receiving support from the state (22). The Medical Research Council recommends the use of these study designs for inferring causality with respect to population health interventions, reflecting the cost, ethical challenges, and practical difficulty of undertaking randomized controlled trials (23, 24). Natural experiments are able to address some of the problems that plague observational studies, on which most of the existing evidence relies (25, 26). Observational studies often lack a well-specified counterfactual condition (i.e., what would have occurred in the absence of an intervention) and are frequently subject to unmeasured confounding (26). Well-conducted randomized controlled trials are the preferable way to establish causality, but there has been conceptual or financial reluctance to use them to evaluate large-scale policies designed to influence the social determinants of health (27). This natural experiment enabled us to address the limitations of both observational studies and randomized controlled trials and therefore estimate the causal relationships between reductions in income, unaffordable housing, and mental ill health.

The health effects of HB reductions are likely to vary across local authorities and will be particularly large in urban areas, where rents are high and where the proportion of HB recipients is large (15). For example, the April 2011 changes reduced the level of financial support by 10% in some parts of Wales but by as much as 50% in some parts of London, where housing costs are much higher. More deprived areas are also likely to be hit harder, as they have a greater proportion of HB recipients.

Here, we tested the association between a reduction in income and mental health by examining changes in the HB, using a control group of persons who were living in privately rented housing but not receiving the HB and an intervention group of otherwise similar individuals who were receiving the HB. We also explored whether the impact of these reductions in the HB was larger in areas hardest hit by this policy change.

## METHODS

### Data

The Annual Population Survey (APS) is a nationally representative, repeated cross-sectional survey of approximately 320,000 individuals conducted annually in the United Kingdom between April and the following March (e.g., April 2010 to March 2011) (28). The APS integrates the Labour Force Survey (waves 1 and 5), the English Local Labour Force Survey, the Welsh Labour Force Survey, and the Scottish Labour Force Survey. These data generate quarterly official statistics, so each quarter is intended to be nationally representative. Details on the survey have been provided elsewhere (28). Briefly, 16,640 households are randomly selected from the “small users” postcode address file. To ensure that no particular household is interviewed too frequently (thus overburdening that household), a “used address file” is maintained, and any address sampled will not be sampled again during the subsequent 2 years. Data are collected through face-to-face interviews. Data were drawn from April 2009 to March 2013, thereby including data from 4 full waves of the survey. These data allowed us to observe mental health at baseline during the preintervention period and then to observe the level of mental ill health after the policy change was made in April 2011. Our analysis focused on persons aged 16–69 years who were then renting housing in the private sector (*n* = 179,037).

### Mental health outcomes

Health is assessed in the APS by asking respondents what health problems they currently have. The list can include physical problems or disabilities connected with the arms, legs, or back. We were principally interested in those people who self-reported “depression, bad nerves, or anxiety” (see the Web Appendix, available at http://aje.oxfordjournals.org/).

Respondents are also asked whether they receive any state benefits, and we recorded whether they self-reported being a recipient of the HB. We also created indicators capturing whether recipients claimed the job-seeker's allowance (unemployment insurance) or—as a sensitivity test—whether they received child tax credits (means-tested financial support for primary caregivers). The APS also collects data on a series of sociodemographic characteristics, including age, sex, government office region (a geographical indicator), ethnicity, number of dependent children, marital status, earnings, employment status, occupation (in detailed occupational categories), education, and disability status.

### Natural experiment study design

We compared people who were renting housing in the private sector and *not* receiving the HB with persons who were also renting in the private sector but *were* receiving the HB (24, 25). We followed these 2 groups before and after the HB policy change was implemented in April 2011, comparing the period April 2009–March 2011 with the period April 2011–March 2013. One key assumption was that assignment to the intervention group should have been random or “as if” random. In this study, we assumed that collection of the APS data was not causally related to the policy change (i.e., who was sampled and when they were sampled) (21). Proceeding from this assumption, we argue that those surveyed prior to the April 2011 reform comprised one nationally representative sample (*n* = 85,090) and those surveyed after the April 2011 reform comprised another nationally representative sample (*n* = 93,974). The only substantive difference between these 2 samples should have been the policy change related to the private rental sector.

To test this assumption, we used a series of balance tests which sought to establish whether the pre–policy change and post–policy change samples were similar across a range of sociodemographic characteristics (Table 1) (22). We examined whether the intervention was random with respect to the sample—that is, whether the policy change was an exogenous change. There were significant (*P* < 0.05) differences for age, ethnicity, number of job-seeker's allowance claimants, gross weekly earnings, employment status, education, disability, and the proportion of the sample who were claiming the HB, but in each case the difference was not substantially important. For example, the proportion of the sample who reported being nonwhite British was 1 percentage point higher in the postintervention sample.

**Table 1.** Tests of Balance Between Study Periods (Comparing April 2009–March 2011 With April 2011–March 2013) for Data From the United Kingdom's Annual Population Survey

Variable	Study Period		Difference (After − Before)	*P* Value^a^
	Before April 2011 (*n* = 85,090)	After April 2011 (*n* = 93,974)		
Sex (female = 1)	0.53 (0.0017)^b^	0.53 (0.0016)	0.0023 (0.0024)	0.34
Age, years	36.97 (0.049)	37.17 (0.067)	0.20 (0.067)	0.0031
London (vs. all other areas)	0.14 (0.0012)	0.14 (0.0011)	0.0008 (0.0016)	0.63
Ethnicity (nonwhite British = 1)	0.14 (0.0012)	0.15 (0.0017)	0.011 (0.0017)	<0.0001
JSA claimant (yes = 1)	0.052 (0.0008)	0.055 (0.007)	0.0034 (0.0011)	0.0015
Marital status (not married = 1)	0.52 (0.0017)	0.51 (0.0016)	0.0008 (0.0024)	0.75
Gross weekly earnings,^c^ £	360.41 (1.82)	378.08 (2.22)	17.67 (3.09)	<0.0001
Employment status (employed = 1)	0.55 (0.0017)	0.56 (0.0016)	0.0147 (0.0024)	<0.0001
Education (NQF level 4 = 1)	0.22 (0.0014)	0.26 (0.0014)	0.039 (0.0020)	<0.0001
Disability (disabled = 1)	0.25 (0.0015)	0.25 (0.0014)	-0.0003 (0.0020)	0.90
Housing benefit (yes = 1)	0.20 (0.0014)	0.21 (0.0013)	0.014 (0.0019)	<0.0001

Abbreviations: JSA, job-seeker's allowance; NQF, National Qualifications Framework.

^a^ *P* value was calculated using a 2-tailed *t* test assuming unequal variances.

^b^ Values are presented as mean (standard error) and are probabilities unless otherwise specified.

^c^ The gross weekly earnings variable was restricted to persons who were employed; therefore, the sample size was smaller (before April 2011: *n* = 32,417; after April 2011: *n* = 36,212). Dollar equivalents: before April 2011—$470.08 (2.37); after April 2011—$493.13 (2.90); difference—$23.05 (4.03).

To estimate the effect of the policy change on mental health, we used difference-in-differences models, which measured the change in mental health over time in both the intervention group and the control group (19). One strength of this econometric technique is that it mimics an experimental research design using observational data by estimating the effect of an intervention on an outcome by comparing the average change in the intervention group with the average change in the nonintervention group. Ideally, repeated observations would be measured within the same individuals over time, but this technique is widely used with repeated cross-sectional data (19).

(1) $$\begin{array}{rl} \text{Intervention}\;\text{effect}=\; & \left({\bar{\text{Depression}}}_{\mathrm{Post},\;\mathrm{HB}}-{\bar{\text{Depression}}}_{\mathrm{Pre},\;\mathrm{HB}}\right)\\ & -({\bar{\text{Depression}}}_{\mathrm{Post},\;\mathrm{non}\text{-}\mathrm{HB}}\\ & -{\bar{\text{Depression}}}_{\mathrm{Pre},\;\mathrm{non}\text{-}\mathrm{HB}}). \end{array}$$

$\bar{\text{Depression}}$ is a measure of the average level of self-reported depressive symptoms and similar mental health problems. The subscript “Post” refers to those people interviewed after the policy change in April 2011, and the subscript “Pre” refers to those interviewed before the policy change. The subscript “HB” indicates persons who are recipients of the HB, and the subscript “non-HB” indicates those who are not recipients of the HB. The intervention effect is our coefficient of interest and is the difference between these 2 observed effects for recipients and nonrecipients, yielding the main difference-in-differences estimator (19). We fitted our models using a linear probability model with robust standard errors to avoid some of the problems associated with logistic and probit regression models; but as a sensitivity test, we ensured that our results were consistent when assuming this alternative functional form (29).

To further check the sensitivity of our findings to our model specification, we also fitted these models using interrupted time-series analysis and used matching methods to observe whether our results were consistent across specifications (25, 30).

## RESULTS

### Mental health impact of HB reductions

**Figure 1.**
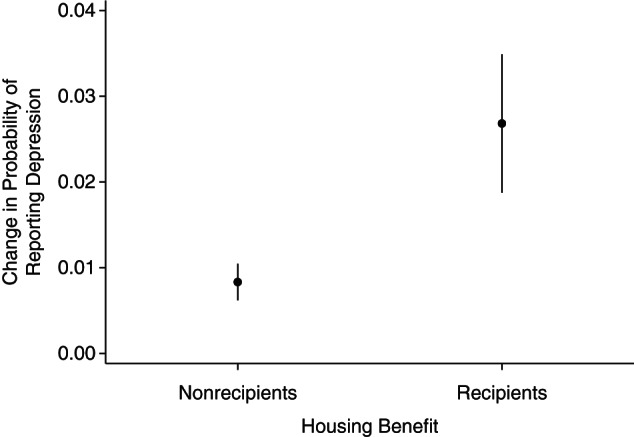
Association between Housing Benefit reform and mental health between April 2009 and March 2013 among private renters in the United Kingdom. The graph shows the change in the probability of reporting depression for recipients and nonrecipients of the government Housing Benefit from a difference-in-differences model, comparing April 2009–March 2011 with April 2011–March 2013. Data were obtained from the Annual Population Survey. The model adjusted for age, sex, employment status, geographical region, ethnicity, number of dependent children in the household under the age of 19 years, income, occupation, education, and whether the respondent was a job-seeker's allowance claimant.

Table 2 shows trends in the prevalence of depressive symptoms, before and after the HB reductions in April 2011. There was a marked increase in the prevalence of depressive symptoms among HB recipients (0.028 percentage points; 95% confidence interval (CI): 0.019, 0.035). In contrast, persons who were not receiving the HB (the “control group”) experienced only a slight, albeit also significant, increase in the probability of self-reported symptoms of depression (0.0083 percentage points; 95% CI: 0.0062, 0.010). This resulted in an inequality in their trends of 1.8 percentage points (95% CI: 0.010, 0.027) (Figure 1).

**Table 2.** Association Between Housing Benefit Reform and Mental Health Among Private Renters in the United Kingdom Between April 2009 and March 2013^a^

	Probability (SE) of People Reporting Depression	
	Model 1: No Covariates	Model 2:^b^ All Covariates
Difference-in-difference estimate (after April 2011)	0.013^c^ (0.0044)	0.018^c^ (0.0043)
Change over time (before April 2011 and after April 2011)	0.0058^c^ (0.0011)	0.0083^c^ (0.0011)
Difference between HB recipients and non-HB recipients before April 2011	0.16^c^ (0.0032)	0.11^c^ (0.0032)
Constant (probability of depression among non-HB recipients before April 2011)	0.042^c^ (0.00077)	0.049^c^ (0.0085)
No. of observations	179,064	179,037

Abbreviations: HB, Housing Benefit; SE, standard error.

^a^ Data were obtained from the United Kingdom's Annual Population Survey. The period April 2009–March 2011 (before reform) was compared with the period April 2011–March 2013 (after reform).

^b^ The following control variables were included in model 2: age, sex, employment status, geographical region, ethnicity, number of dependent children in the household under the age of 19 years, income, occupation, education, and whether the respondent was a job-seeker's allowance claimant.

^c^ *P* < 0.01.

**Figure 2.**
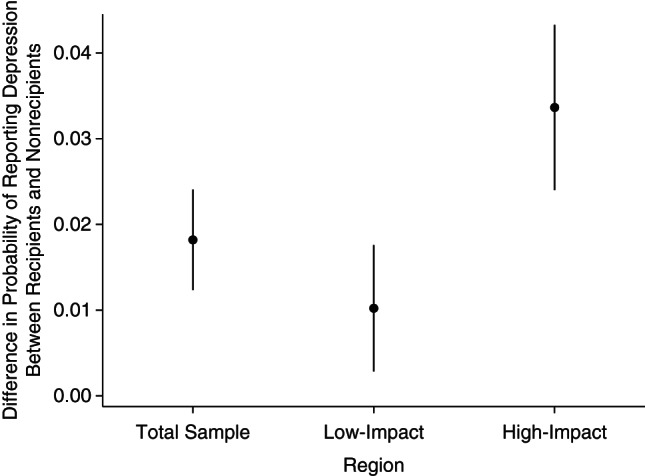
Association between Housing Benefit reform and mental health between April 2009 and March 2013 among private renters in the United Kingdom, by region of impact (total sample, low-impact areas, and high-impact areas). The graph shows difference-in-differences estimates of change in the probability of reporting depression for recipients and nonrecipients of the Housing Benefit, comparing April 2009–March 2011 with April 2011–March 2013. Data were obtained from the Annual Population Survey. The models adjusted for age, sex, employment status, geographical region, ethnicity, number of dependent children in the household under the age of 19 years, income, occupation, education, and whether the respondent was a job-seeker's allowance claimant.

Some areas of the country were hit harder by the changes than others (15, 18). Drawing on simulations of the effects of this change in the HB, we would expect these reforms to increase financial strain more dramatically in the most expensive housing markets, such as Inner London, the South East, and other major urban areas. We calculated separate difference-in-differences estimates for persons living in high- and low-impact areas. We found that the increase in depressive symptoms was greater among private renters receiving the HB in high-impact areas than among HB recipients in low-impact areas (Figure 2). However, even in these low-impact areas, the prevalence of depressive symptoms rose approximately 1 percentage point more than for private renters who were not receiving the HB.

### Alternative estimates of the influence of welfare reform on mental health

**Figure 3.**
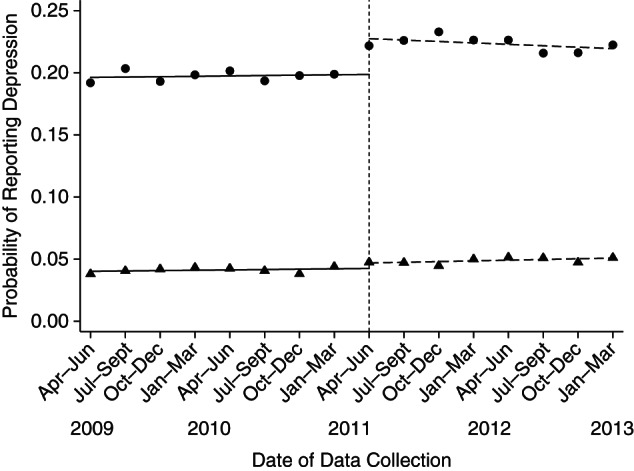
Interrupted time-series analysis of the association between Housing Benefit (HB) reform and mental health between April 2009 and March 2013 among private renters in the United Kingdom. The analysis examined quarterly estimates from the Annual Population Survey, comparing April 2009–March 2011 with April 2011–March 2013. The vertical dashed line represents implementation of the change in the government HB in April 2011. The graph shows the probability of reporting depression among recipients (circles) and nonrecipients (triangles) of the HB. The solid black lines show the trend in the probability of reporting depression before April 2011 for both HB recipients and nonrecipients. The dashed black lines show the trend in the probability of reporting depression after April 2011 for both HB recipients and nonrecipients.

To ensure that our results were not solely due to our modeling strategy, we also estimated the influence of this policy intervention on mental ill health using interrupted time-series analysis (25). To do this, we calculated the prevalence of self-reported depressive symptoms for each quarter among people in the private rental sector who did and did not receive the HB. Then we estimated whether there was a break in the aggregate trend around April 2011 for persons receiving the HB compared with those who were not receiving the HB. This form of analysis allowed us to test whether the parallel trends assumption held (i.e., the trajectories for the intervention and control groups would have been the same in the absence of the policy change) (19). Consistent with the difference-in-differences models, we found that there was a clear break in the data around April 2011 for persons receiving the HB over and above any change seen among those who were not receiving the HB (Figure 3; Web Table 1). In addition, we found that the trends for both groups before the intervention were parallel and that there was no clear break in the control group after the policy change, which supports the parallel trends assumption. We also found no evidence that the influence of the reform diminished over time, indicating that the health effects of increasing financial strain among recipients of the HB did not attenuate quickly as people adjusted to this new reality.

Finally, because people receiving the HB differ in important ways from those not receiving the HB, we implemented a matching procedure (using coarsened exact matching) to balance the sample across a range of attributes, except (importantly) whether respondents received the HB (30, 31). We matched on the following covariates: age, sex, employment status, geographical region, ethnicity, number of dependent children in the household under the age of 19 years, income, occupation, education, whether the respondent was a job-seeker's allowance claimant, and the date of interview (see Web Table 2 for more details on the adequacy of the matching procedure). We found that even after matching, the change in the prevalence of depressive symptoms among HB recipients remained larger than the change among private renters who were not HB recipients, even though the estimated effect was attenuated slightly (Table 3).

**Table 3.** Association Between Housing Benefit Reform and Mental Health Among Private Renters in the United Kingdom (Matching Model) Between April 2009 and March 2013^a^

	Probability (SE) of People Reporting Depression^b^
Difference-in-difference estimate (after April 2011)	0.011^c^ (0.0038)
Change over time (before April 2011 and after April 2011)	0.0086^c^ (0.0019)
Difference between HB recipients and non-HB recipients before April 2011	0.12^d^ (0.0028)
Constant (probability of depression among non-HB recipients before April 2011)	0.078 (0.0014)
No. of observations	150,731

Abbreviations: HB, Housing Benefit; SE, standard error.

^a^ Data were obtained from the United Kingdom's Annual Population Survey. The period April 2009–March 2011 (before reform) was compared with the period April 2011–March 2013 (after reform).

^b^ Control variables included age, sex, employment status, geographical region, ethnicity, number of dependent children in the household under the age of 19 years, income, occupation, education, and whether the respondent was a job-seeker's allowance claimant.

^c^ *P* < 0.01.

^d^ *P* < 0.001.

### Robustness tests

We performed a series of robustness checks. First, we conducted 2 so-called “falsification tests,” which checked the specificity of our findings. In one test, we examined whether symptoms of depression changed among HB recipients living in local authority (government-owned) housing, who would not have been affected by adjustments to the private sector housing allowance (*n* = 76,467) (32); we found that there was no discernible effect in this group (Table 4). In a further test, we assessed physical health outcomes that would not plausibly change in the short term, such as “difficulty in hearing” or a “speech impediment” (Web Appendix). As expected, no relationship was observed (Table 5), further corroborating the clinical relevance of our observations.

**Table 4.** Analysis of Whether the Influence of Housing Benefit Reform in the United Kingdom Varied by Rental Sector Between April 2009 and March 2013^a^

	Probability (SE) of People Reporting Depression^b^	
	Model 1: Local Authority Housing	Model 2: Private Rental Sector
Difference-in-difference estimate (after April 2011)	0.010 (0.0059)	0.018^c^ (0.0043)
Change over time (before April 2011 and after April 2011)	0.018^c^ (0.0023)	0.0083^c^ (0.0011)
Difference between HB recipients and non-HB recipients before April 2011	0.12^c^ (0.0043)	0.11^c^ (0.0032)
Constant (probability of depression among non-HB recipients before April 2011)	0.12^c^ (0.014)	0.049^c^ (0.0085)
No. of observations	76,467	179,037

Abbreviations: HB, Housing Benefit; SE, standard error.

^a^ Data were obtained from the United Kingdom's Annual Population Survey. The period April 2009–March 2011 (before reform) was compared with the period April 2011–March 2013 (after reform).

^b^ Control variables for both models included age, sex, employment status, geographical region, ethnicity, number of dependent children in the household under the age of 19, income, occupation, education, and whether the respondent was a job-seeker's allowance claimant.

^c^ *P* < 0.01.

**Table 5.** Association Between Housing Benefit Reform and Other Health Problems Among Private Renters in the United Kingdom Between April 2009 and March 2013^a^

	Probability (SE) of People Reporting Other Health Challenges	
	Model 1: No Covariates	Model 2:^b^ All Covariates
Difference-in-difference estimate (after April 2011)	−0.013^c^ (0.0063)	−0.00067 (0.0058)
Change over time (before April 2011 and after April 2011)	−0.0054^c^ (0.0023)	−0.0016 (0.0022)
Difference between HB recipients and non-HB recipients before April 2011	0.20^d^ (0.0046)	0.090^d^ (0.0045)
Constant (probability of depression among non-HB recipients before April 2011)	0.24^d^ (0.0017)	0.048^d^ (0.016)
No. of observations	164,839	164,814

Abbreviations: HB, Housing Benefit; SE, standard error.

^a^ Data were obtained from the United Kingdom's Annual Population Survey. The period April 2009–March 2011 (before reform) was compared with the period April 2011–March 2013 (after reform).

^b^ The following control variables were included in model 2: age, sex, employment status, geographical region, ethnicity, number of dependent children in the household under the age of 19 years, income, occupation, education, and whether the respondent was a job-seeker's allowance claimant.

^c^ *P* < 0.05.

^d^ *P* < 0.01.

In the APS, there is also an alternative measure of mental health problems which includes “phobias” and “panic attacks.” We included these people in our main “depression” dependent variable to see whether our results changed, finding that they did not (Web Table 3). Next, we tested whether reductions to child tax credits, which also began around April 2011, might have influenced our results. We found that including this additional policy change in the model did not qualitatively alter our findings (Web Table 4). We also removed people from the sample who had had preexisting mental health problems for over 1 year (*n* = 911), as well as those who had any health problem (*n* = 6,289). As shown in Web Table 5, none of the results changed. This further supports the conclusion that the change in prevalence corresponded to new cases of depressive symptoms (Web Table 5).

Although there was a “used address file,” it is possible that the same individuals were sampled twice. Therefore, we also removed any duplicate observations for which the same individual may have been sampled twice in 2 different periods, finding that our results remained unchanged (Web Table 6). We compared the influence of the April 2011 reforms in the HB with the whole sample (i.e., we compared people receiving the HB in the private rental sector with everyone else in the data set) and found that our results did not qualitatively change (Web Table 7). Respondents who report depressive symptoms are highly likely to report being disabled. We therefore adjusted our models for disability status and observed that our results remained consistent across these models (Web Table 8). We also estimated our main associations using a logistic regression model and found similar results (Web Figure 1).

## DISCUSSION

The reforms made to the LHA in 2011 created a natural experiment which reduced financial support for one group in the private rental sector (renters who received the HB) but not others (renters who did not receive the HB). Using difference-in-differences models, interrupted time-series analysis, and matching methods, we consistently found that the April 2011 reforms to the HB increased the risk of depressive symptoms among persons claiming the HB by approximately 1.8 percentage points above the risk in private renters not claiming the benefit. In short, our results indicate that a reduction in household income (which reduced the level of housing affordability) harmed mental health. This rise in mental ill health was not only a short-term shock; the increase in depressive symptoms remained elevated for up to 24 months after the reform (see Figure 3). In addition, the rise in symptoms of depression among persons in the private rental sector was concentrated in those regions of the United Kingdom hit hardest by the reforms, such as Inner London and Tyne and Wear.

To put this rise in perspective, in March 2010—just before the reform—there were 1.5 million people receiving the HB in the private rental sector. Approximately 20% of these people were experiencing depression. After the reform, prevalence of this self-reported measure of poor mental health rose by 1.8 percentage points—roughly a 10% increase. According to our models, approximately 26,000 (95% confidence interval: 14,000, 38,000) additional HB recipients reported symptoms of depression after these reforms to the HB occurred.

Our study had several limitations. First, we used repeated cross-sectional survey data, so it is not entirely clear whether those who reported symptoms of depression after the 2011 reform were new claimants or existing claimants. However, our results indicate that the characteristics of the sample were very similar before and after the intervention, suggesting that compositional changes do not explain these findings. Further, these findings are consistent with longitudinal qualitative data collected on those experiencing the reforms (33). Second, we cannot know what would have happened to the level of depressive symptoms in the intervention group if the reform had not been implemented. To address this, we examined the parallel trends assumption and observed that the trajectories of depressive symptoms among the intervention and control groups were parallel before April 2011, suggesting that, in the absence of the intervention, these trends would have continued to be parallel (25). Third, other policy changes which may have influenced mental health were occurring during this same period, such as changes to child tax credits. Even when we adjusted for these policy changes we found that our results remained consistent, suggesting that these findings are not explained by other policy reforms.

Fourth, it is possible that HB recipients may have stated that they were not receiving the HB because they were embarrassed to admit they were claiming state support, thereby potentially biasing our results. However, such underreporting would have led to conservative estimates, because those most at risk of experiencing depressive symptoms following April 2011 were now included in the control group, narrowing the gap between the intervention and control groups. Fifth, the timing of the April 2011 changes in the LHA may not have been “as if” random, because people in the intervention group were aware of the policy change and may have adjusted their behavior beforehand, potentially undermining our ability to detect an effect. This suggests that our estimates of the causal effect of reduced income on the affordability of housing and mental health are likely to be conservative. Sixth, people in the private rental sector may also be experiencing poorer mental health because they are struggling to obtain a mortgage, something that would have been a more realistic prospect 20 years ago. The impact of the changing prospects of housing ownership on mental health requires detailed further study, but is unlikely to have affected our results because those prospects were relatively stable across this period. Seventh, our measure of depressive symptoms was a self-report measure, and although self-reporting is correlated with clinical assessments, it is not itself a diagnostic tool. It may therefore have biased our results, particularly among men, who may be less likely to report symptoms of depression in this survey setting, creating conservative biases (34).

These results have important policy implications. First, they highlight the health effects of welfare reform on economically vulnerable groups (35). These changes to the private rental sector have reduced financial support for these groups, increasing the precariousness of their housing situations and exposing them to greater insecurity and potentially homelessness (36). Second, these reforms to the LHA (by increasing the level of depressive symptoms among recipients of the HB) potentially counteract policy initiatives in other areas that were designed to reduce reliance on disability benefits by reducing the generosity of incapacity benefits. In the future, policy-makers should attend more carefully to the spillover effects of policy interventions that are implemented simultaneously. Similarly, the costs incurred in treating persons whose mental health has deteriorated may offset any potential savings resulting from cutting the HB. Third, as part of the July 2015 budget, the Conservative government outlined plans to remove eligibility for the HB from youths aged 18–21 years. Our results suggest that this policy change, if implemented as scheduled in 2017, will increase the risk of depressive symptoms in this age group even further, potentially harming their chances in the labor market and having a long-term scarring effect on their health.

## Supplementary Material

Web Material
