# Supplementary material for: Reductions in the United Kingdom's Government Housing Benefit and Symptoms of Depression in Low-Income Households
Source: Am J Epidemiol. 2016 Sep 8;184(6):421–9. doi: 10.1093/aje/kww055 (PMC5023793; doi:10.1093/aje/kww055)
Supplement: Web Material [file supp_184_6_421__index.html]

Reductions in the United Kingdom's Government Housing Benefit and Symptoms of Depression in Low-Income Households — Web Material 

# Reductions in the United Kingdom's Government Housing Benefit and Symptoms of Depression in Low-Income Households

## Web Material

Web Material

- Web Material - Pdf file
