# Supplementary material for: Reductions in the United Kingdom's Government Housing Benefit and Symptoms of Depression in Low-Income Households
Source: Am J Epidemiol. 2016 Sep 8;184(6):421–9. doi: 10.1093/aje/kww055 (PMC5023793; doi:10.1093/aje/kww055)
Supplement: Web Material [file supp_kww055_kww055supp.pdf]

Web Materials for "Reductions in the United Kingdom's Government Housing Benefit and Symptoms of Depression in Low-Income United Kingdom Households" by Reeves et al.

Web Appendix. Question assessing health in the United Kingdom's Annual Population Survey

Web Table 1. Interrupted time series analysis of housing benefit reform and mental health between April 2009 and March 2013, private renters

Web Table 2. Details of the coarsened exact matching model

Web Table 3. Housing benefit reform and mental health (including phobias) between April 2009 and March 2013, private renters

Web Table 4. Housing benefit reform and mental health between April 2009 and March 2013, private renters, adjusting for changes in child tax credits

Web Table 5. Housing benefit reform and mental health between April 2009 and March 2013, private renters, excluding people with past depression problems and those with pre-existing health problems

Web Table 6. Housing benefit reform and mental health between April 2009 and March 2013, private renters, removing duplicate observations

Web Table 7. Housing benefit reform and mental health is main health problem between April 2009 and March 2013, all people

Web Table 8. Housing benefit reform and mental health between April 2009 and March 2013, private renters, adjusting for disabled status

Web Figure 1. Housing benefit reform and mental health between April 2009 and March 2013, private renters, logistic regression model

Web Appendix. Question assessing health in the United Kingdom's Annual Population Survey

What health problems does the respondent have:

(1) Problems or disabilities (including arthritis or rheumatism) connected with... arms or hands
(2) ....legs or feet
(3) ....back or neck
(4) Difficulty in seeing (while wearing spectacles or contact lenses)
(5) Difficulty in hearing
(6) A speech impediment
(7) Severe disfigurement, skin conditions, allergies
(8) Chest or breathing problems, asthma, bronchitis
(9) Heart, blood pressure or blood circulation problems
(10) Stomach, liver kidney or digestive problems
(11) Diabetes
(12) Depression, bad nerves or anxiety
(13) Epilepsy
(14) Severe or specific learning difficulties (mental handicap)
(15) Mental illness, or suffer from phobia, panics or other nervous disorders
(16) Progressive illness not included elsewhere (e.g., cancer, multiple sclerosis, symptomatic HIV, Parkinson's disease, muscular dystrophy)
(17) Other health problems or disabilities

Web Table 1. Interrupted time series analysis of housing benefit reform and mental health between April 2009 and March 2013, private renters

	**Probability of People Reporting Depression (Model 1)**
Difference in the change in the probability for the intervention and control groups after April 2011	-0.0017 (0.0011)
Difference between intervention and control groups after April 2011	0.024*** (0.0050)
Change in the probability of depression among control group after April 2011 (pre-intervention slope)	0.0003 (0.0005)
Increase in the mean probability of reporting depression after April 2011 among control group	0.0044 (0.0024)
Difference in the change in the probability for the intervention and control groups before April 2011	0.0000 (0.0008)
Difference between intervention and control groups before April 2011 (pre-intervention constant)	0.16*** (0.0045)
Change in the probability of depression among control group before April 2011 (pre-intervention slope)	0.0003 (0.0004)
Mean probability of reporting depression before April 2011 among non-HB group (constant)	0.040 (0.0016)
No. of observations	32

Source: Annual Population Survey. Newey-West standard errors are reported in parentheses.
HB, housing benefit.
* $P < 0.05$ ** $P < 0.01$ *** $P < 0.001$

Web Table 2. Details (mean (SE)) of the coarsened exact matching model

**Variable**	**Not Receiving Housing Benefit (*n* = 142,205)**	**Receiving Housing Benefit (*n* = 36,859)**	**Difference (After − Before)**	***P* Value**
Sex (female = 1)	0.65 (0.0022)	0.65 (0.0025)	<0.0001 (0.0033)	0.99
Age, years	41.93 (0.064)	42.33 (0.098)	-0.41 (0.098)	<0.0001
London	0.11 (0.0017)	0.11 (0.0016)	<0.0001 (0.0023)	0.99
Ethnicity (non-white British = 1)	0.12 (0.0016)	0.12 (0.0017)	<0.0001 (0.0023)	0.99
JSA claimant	0.13 (0.0024)	0.013 (0.0017)	<0.0001 (0.0029)	0.99
Marital status (not married = 1)	0.47 (0.0024)	0.47 (0.0026)	<0.0001 (0.0036)	0.99
Education (NQF level 4 = 1)	0.10 (0.0013)	0.10 (0.0016)	<0.0001 (0.0021)	0.99

JSA, job-seeker's allowance; NQF, National Qualifications Framework; SE, standard error.

P value was calculated using a two-tailed *t* test.

The matching variables included: age, sex, employment status, geographical region, ethnicity, number of dependent children in household under the age of 19, income, occupation, education, whether the respondent was a JSA claimant, and the date of interview. Pre-matching models contain 179,064 observations while the matched model only contains 150,731 observations. Therefore, 28,333 observations were trimmed from the sample because there were no matches available for these individuals. Almost all of these 28,333 observations (98.25%) were non-Housing Benefit recipients. These trimmed observations are observations that are potentially creating imbalances in the main pre-matched models. Using the coarsened exact matching procedure we can calculate an imbalance statistic (1 = complete imbalance or complete separation, 0 = complete balance) (1,2). Before matching the imbalance statistic is 0.53 but after performing the matching procedure the imbalance statistic is <0.01, suggesting that the degree of balance in the sample is nearly perfect (see table below). In short, the matching procedure addresses many of the differences that might bias the results between those who receive housing benefits and those who do not.

Balance tests after matching procedure for those claiming housing benefit and those not claiming housing benefit.

Web Table 3. Housing benefit reform and mental health (including phobias) between April 2009 and March 2013, private renters

	**Probability of People Reporting Depression or Phobias (Model 1)**
Difference-in-difference estimate (after April 2011)	0.019*** (0.0044)
Change over time (before April 2011 and after April 2011)	0.0085** (0.002)
Difference between HB recipients and non-HB recipients before April 2011	0.12*** (0.0034)
Constant (probability of depression among non-HB recipients before April 2011)	0.070 (0.0094)
No. of observations	179,037
R^2	0.13

Source: Annual Population Survey. Robust standard errors are reported in parentheses. Control variables include: age, sex, employment status, geographical region, ethnicity, number of dependent children in household under the age of 19, income, occupation, education, and whether the respondent was a JSA claimant.

HB, housing benefit; JSA, job-seeker's allowance.

* $P < 0.05$ ** $P < 0.01$ *** $P < 0.001$

Web Table 4. Housing benefit reform and mental health between April 2009 and March 2013, private renters, adjusting for changes in child tax credits

	**Probability of People Reporting Depression (Model 1)**
Difference-in-difference estimate (after April 2011)	0.019*** (0.0043)
Change over time (before April 2011 and after April 2011)	0.0086*** (0.0011)
Difference between HB recipients and non-HB recipients before April 2011	0.11*** (0.0032)
Constant (probability of depression among non-HB recipients before April 2011)	0.049 (0.0085)
No. of observations	179,037
R^2	0.11

Source: Annual Population Survey. Robust standard errors are reported in parentheses. Control variables include: age, sex, employment status, geographical region, ethnicity, number of dependent children in household under the age of 19, income, occupation, education, and whether the respondent was a JSA claimant.

HB, housing benefit; JSA, job-seeker's allowance.

This model also includes a measure of whether respondents receive child tax credits or not and an interaction term between child tax credits and time, to capture any effect of these changes on health.

* $P < 0.05$ ** $P < 0.01$ *** $P < 0.001$

Web Table 5. Housing benefit reform and mental health between April 2009 and March 2013, private renters, excluding people with past depression problems and those with pre-existing health problems

	**Probability of People Reporting Depression (Excluding People With Past Depression) (Model 1)**	**Probability of People Reporting Depression (Excluding People With Past Health Problems) (Model 3)**
Difference-in-difference estimate (after April 2011)	0.018*** (0.0043)	0.018** (0.0044)
Change over time (before April 2011 and after April 2011)	0.0085*** (0.0011)	0.0087** (0.0011)
Difference between HB recipients and non-HB recipients before April 2011	0.11*** (0.0032)	0.11** (0.0033)
Constant (probability of depression among non-HB recipients before April 2011)	0.050 (0.0085)	0.045** (0.0085)
No. of observations	178,126	172,748
R^2	0.11	0.11

Source: Annual Population Survey. Robust standard errors are reported in parentheses. Control variables include: age, sex, employment status, geographical region, ethnicity, number of dependent children in household under the age of 19, income, occupation, education, and whether the respondent was a JSA claimant. This model excludes anyone who reports having depression in the past and those who have other pre-existing health problems.

HB, housing benefit; JSA, job-seeker’s allowance.

* $P < 0.05$ ** $P < 0.01$ *** $P < 0.001$

Web Table 6. Housing benefit reform and mental health between April 2009 and March 2013, private renters, removing duplicate observations

	**Probability of People Reporting Depression (Model 1)**
Difference-in-difference estimate (after April 2011)	0.015** (0.0052)
Change over time (before April 2011 and after April 2011)	0.0078*** (0.0012)
Difference between HB recipients and non-HB recipients before April 2011	0.11*** (0.0041)
Constant (probability of depression among non-HB recipients before April 2011)	0.036 (0.0096)
No. of observations	130,405
R^2	0.11

Source: Annual Population Survey. Robust standard errors are reported in parentheses. Control variables include: age, sex, employment status, geographical region, ethnicity, number of dependent children in household under the age of 19, income, occupation, education, and whether the respondent was a JSA claimant.

HB, housing benefit; JSA, job-seeker’s allowance.

Web Table 7. Housing benefit reform and mental health between April 2009 and March 2013, all people

	**Probability of People Reporting Depression (Model 1)**
Difference-in-difference estimate (after April 2011)	0.016***<br>(0.0033)
Change over time (before April 2011 and after April 2011)	0.0093***<br>(0.0004)
Difference between HB recipients and non-HB recipients before April 2011	0.15***<br>(0.0024)
Constant (probability of depression among non-HB recipients before April 2011)	0.069<br>(0.0042)
No. of observations	867,558
R^2	0.075

Source: Annual Population Survey. Robust standard errors are reported in parentheses. Control variables include: age, sex, employment status, geographical region, ethnicity, number of dependent children in household under the age of 19, income, occupation, education, and whether the respondent was a JSA claimant.

HB, housing benefit; JSA, job-seeker's allowance.

* $P < 0.05$ ** $P < 0.01$ *** $P < 0.001$

Web Table 8. Housing benefit reform and mental health between April 2009 and March 2013, private renters, adjusting for disabled status.

	**Probability of People Reporting Depression (Model 1)**
Difference-in-difference estimate (after April 2011)	0.016***<br>(0.0039)
Change over time (before April 2011 and after April 2011)	0.0079***<br>(0.0010)
Difference between HB recipients and non-HB recipients before April 2011	0.071***<br>(0.0030)
Constant (probability of depression among non-HB recipients before April 2011)	0.034<br>(0.0078)
No. of observations	179,037
R^2	0.24

Source: Annual Population Survey. Robust standard errors are reported in parentheses. Control variables include: age, sex, employment status, geographical region, ethnicity, number of dependent children in household under the age of 19, income, occupation, education, and whether the respondent was a JSA claimant. The model also adjusted for self-reported disability.

HB, housing benefit; JSA, job-seeker's allowance.

* P < 0.05 ** P < 0.01 *** P < 0.001

Web Figure 1. Housing benefit reform and mental health between April 2009 and March 2013, private renters, logistic regression model

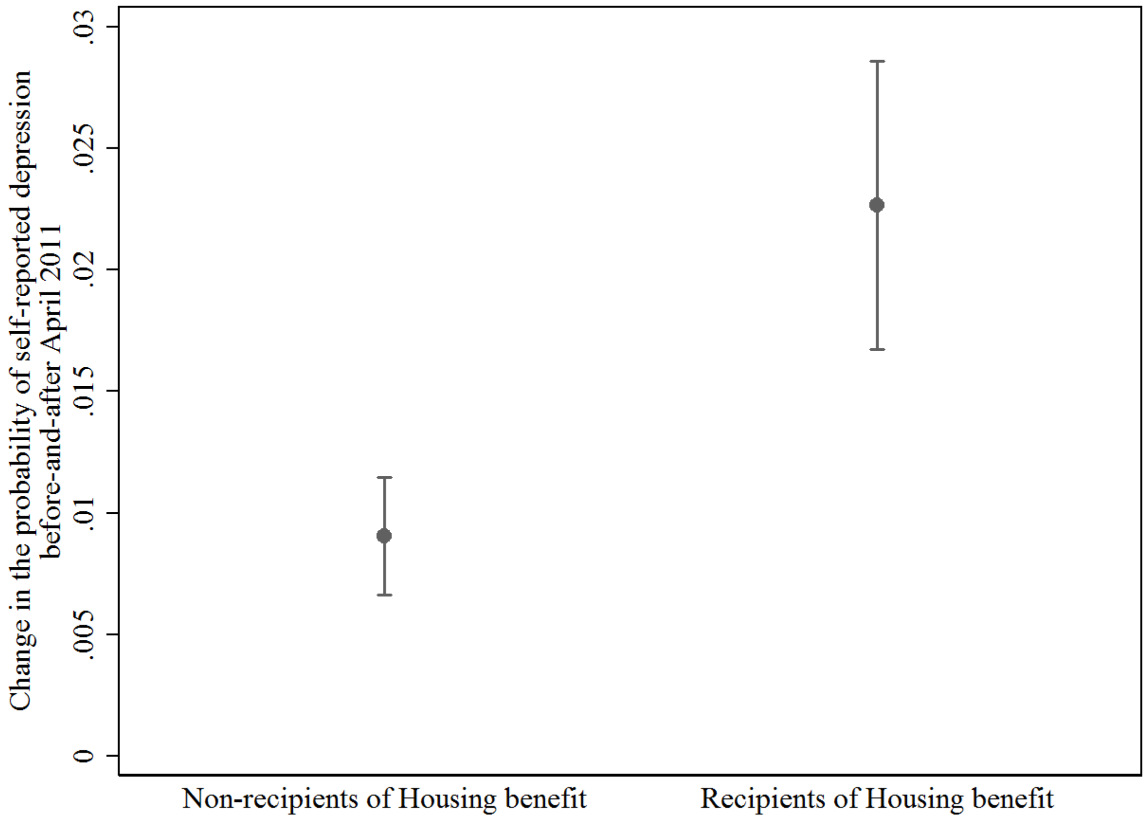

Source: Annual Population Survey. Robust standard errors are reported in parentheses. Control variables include: age, sex, employment status, geographical region, ethnicity, number of dependent children in household under the age of 19, income, occupation, education, and whether the respondent was a job-seeker's allowance claimant.

Web References

1. Iacus SM, King G, Porro G. Causal inference without balance checking: coarsened exact matching. *Polit Anal.* 2011;mpr013.

2. Iacus SM, King G, Porro G. Multivariate matching methods that are monotonic imbalance bounding. *J Am Stat Assoc.* 2011;106(493):345–361.
